# APS PERSPECTIVES ON CHALLENGES AND STRATEGIES SERVING CLIENTS DURING THE COVID-19 PANDEMIC SURGE

**DOI:** 10.1093/geroni/igac059.1599

**Published:** 2022-12-20

**Authors:** Alyssa Elman, Elaine Gottesman, Lena Makaroun, E-Shien Chang, Sunday Clark, Tony Rosen

**Affiliations:** Weill Cornell Medical College / NewYork-Presbyterian Hospital, New York, New York, United States; New York-Presbyterian Hospital/Weill Cornell Medical Center, New York, New York, United States; VA Pittsburgh Healthcare System, Pittsburgh, Pennsylvania, United States; Weill Cornell Medical College, New York, New York, United States; Weill Cornell Medical College / NewYork-Presbyterian Hospital, New York, New York, United States; Weill Cornell Medical College / NewYork-Presbyterian Hospital, PELHAM, New York, United States

## Abstract

The initial surge of the COVID-19 pandemic and public health measures in response dramatically impacted Adult Protective Services’ (APS) ability to conduct investigations and provide services, requiring agencies to quickly adapt. Our goal was to describe challenges for APS and strategies they developed to respond. We conducted 6 focus groups and 7 interviews during March-April 2021 used a semi-structured topic guide, with 31 participants from APS leadership, supervisors, and case workers in New York City, a community hard hit by the initial COVID surge. Focus groups and interviews were recorded and transcribed, with data analyzed to identify themes. Participants identified 9 major challenges, including: clients using concern about COVID-19 to refuse APS workers’ access to their home, necessity to perform home visits/wellness checks on behalf of other agencies who had suspended home visits, and dependence on in-office activities including receiving paper mail. Participants reported 30 formal and informal strategies adopted to address challenges. These included 13 focusing on maximizing client engagement while maintaining client and APS worker safety, such as: adding a pre-investigation before a home visit to assess COVID-19 risk/exposure, offering clients masks and hand sanitizer to build trust, and close collaboration with other agencies/programs including joint visits. Also, 17 strategies were reported to allow for remote work and support staff, including: modifying processes to replace paper mail with electronic communication/processing, offering counseling services, formally recognizing excellent performance, and leadership reaching out personally to check on staff members. These findings may inform APS planning for future large-scale societal disruptions.

